# Recent Trends in Polymer Membranes: Fabrication Technique, Characterization, Functionalization, and Applications in Environmental Science (Part I)

**DOI:** 10.3390/polym16202889

**Published:** 2024-10-14

**Authors:** Yan Wang, Gang Wei

**Affiliations:** College of Chemistry and Chemical Engineering, Qingdao University, Qingdao 266071, China

## 1. Introduction

Polymer membranes have gained significant attention in recent years due to their pivotal role in addressing various environmental challenges such as water purification, gas separation, and pollutant removal [1,2,3]. The unique properties of polymer membranes, including their nanoporous channels, high selectivity, permeability, and flexible tunability, make them ideal candidates for a wide range of applications in environmental science [4,5].

The environmental applications of polymer membranes include desalination, wastewater treatment, air purification, and the recovery of valuable resources from waste streams [6,7,8]. The development of polymer membranes with advanced properties such as high permeability and selectivity, fouling resistance, and stability under harsh conditions has become crucial to advancing these applications [9]. Firstly, the fabrication strategies of polymer membranes are significant to their functionality and performance. Over the past decade, the membrane fabrication techniques have evolved significantly, incorporating advanced methodologies that enable the control of the membrane porosity, thickness, and surface properties [10,11]. Recent advancements in the fabrication techniques, such as phase inversion, electrospinning, layer-by-layer assembly, and 3D printing, have enabled the development of membranes with tailored structures and functions, as well as enhanced performance [12]. In addition, characterization methods such as spectroscopy, microscopy, and permeability analysis, etc. are essential to understanding the membrane structural, thermal, and mechanical properties; the surface chemistry and hydrophilicity; as well as the transport performance metrics [13]. Modern characterization techniques provide detailed insights into the membrane morphology, permeability, and selectivity, which are critical for optimizing their performance. Moreover, innovative functionalization strategies, including surface modification, the incorporation of functional nanomaterials, polymer blending, and composite formation, have expanded the capabilities of polymer membranes, enabling selective separation, anti-fouling properties, and improved mechanical stability [14,15]. In particular, the developments in bioinspired and biomimetic membranes have opened new possibilities for achieving various function-specific applications [16,17,18]. By incorporating biological molecules such as enzymes, protein, and peptides into polymer membranes, researchers have created multifunctional membranes that exhibited enhanced selectivity and capacity for specific pollutants, toxins, or microbial contaminants [19,20]. As a result, these innovative processes promoted the fabrication and functionalization of advanced polymer membranes, which could be favorably adopted in various environmentally relevant application scenarios, including water treatment and desalination, gas separation, air purification, and resource recovery from waste streams.

In this Special Issue, we expect to collect contributions that focus on (but are not limited to) the design and fabrication, characterizations, structural and functional regulation, as well as emerging applications of various polymer membranes for boosting the utilization of membrane materials in environmental science.

## 2. Overview of the Published Articles

Finally, 13 articles, including 2 reviews and 11 research articles, have been published in the first part of this collection.

In the first review (Contribution 1), the authors discussed the use of poly(arylene ether) (PAE)-based polymer membranes for water purification, particularly in harsh environments. Polymer membranes, such as those based on poly(ether ether ketone) (PEEK), polyethersulfone (PES), and poly(arylene ether nitrile) (PEN), were effective in addressing the challenges posed by pollutants, corrosion, and extreme conditions. This review discussed their advantages, such as chemical stability, heat resistance, and durability, along with their fabrication techniques, including phase separation and electrospinning. They further outlined the modification techniques to enhance the membrane performance, focusing on their applications in oil–water separation, desalination, and wastewater treatment. This review provides useful guidance for readers to understand the construction and functionalization of PAE membranes for environmental science applications.

In another review (Contribution 2), Zhao et al. presented the advances in the preparation and applications of porous poly(lactic acid) (PLA) membranes, which have been increasingly applied in tissue engineering, drug release, and oil–water separation. PLA is a biodegradable and biocompatible polymer derived from renewable resources, making it an environmentally friendly alternative to petroleum-based polymers. This review explored various fabrication techniques, including electrospinning, breath-figure, and phase separation methods, and compared their advantages and limitations. The authors also highlighted the challenges of controlling the pore structure and production efficiency while offering insights into future improvements in PLA membrane technologies.

Heavy metals and organic pollutants pose significant environmental and health risks due to their non-biodegradable nature, leading to accumulation in biological systems. Traditional removal techniques such as adsorption, ion exchange, and membrane filtration often suffer from high operating costs and low efficiency. The study by Khedr et al. (Contribution 3) focused on the removal of a harmful heavy metal, lead (Pb(II)), from industrial wastewater. The authors explored the use of low-density polyethylene (LDPE) films grafted with acrylonitrile (AN) and acrylic acid (AAc) for enhanced Pb(II) adsorption. The grafted polymers were chemically stable and provided a cost-effective, efficient solution for metal ion removal from water environments. Vinasse, a byproduct of bioethanol production, contains high concentrations of phenolic acids and polyphenols, which are difficult to degrade and have phytotoxic effects on ecosystems. In another study (Contribution 4), the potential of biopolymers including cellulose, carboxymethylcellulose, and chitosan for the removal of pollutants from vinasse was explored. Using molecular dynamics simulations, the authors examined how these biopolymers interacted with various organic compounds found in vinasse, particularly focusing on their capacity to adsorb and retain pollutants. The study demonstrates that biopolymers could offer a sustainable and cost-effective solution for vinasse treatment, with significant environmental benefits. The presented results also provide valuable insights into industrial wastewater management with functional biopolymers.

Nanomaterial-based functional membranes have gained significant interest due to their wide applications in the fields of filtration, antibacterial materials, and tissue engineering. Among them, graphene oxide (GO) and its derivatives are particularly valued for their remarkable electrical, mechanical, and thermal properties, along with their intrinsic antibacterial properties. However, the antibacterial effectiveness of GO alone is often limited as compared to traditional antibacterial agents, like silver nanoparticles (AgNPs). He and co-workers explored a green synthetic approach to develop multifunctional hybrid membranes combining GO, peptide nanofibers (PNFs), and AgNPs through a solvent-evaporation method (Contribution 5). The GO nanosheets were functionalized with PNFs to enhance their biocompatibility and dispersity, while providing active sites for the AgNP growth. The integration of these materials improved the overall antibacterial properties through synergistic interactions. The developed functional membranes can be used for various biomedical and environmental applications, demonstrating their excellent antibacterial performance against *E. coli* and *S. aureus*.

Hydrogen gas has become a pivotal focus in the global shift towards green energy solutions, due to its potential as a clean, renewable energy source. Water electrolysis, a process used to split water molecules into hydrogen and oxygen, is key to producing ultra-pure hydrogen in an eco-friendly manner. In particular, the anion exchange membrane (AEM) electrolysis has garnered attention due to its high cost-effectiveness and scalability. Noor Azam et al. investigated the influence of operating parameters such as electrolyte concentration, flow rate, and temperature on the efficiency and hydrogen production performance of AEM electrolysis systems (Contribution 6). The overall objective was to optimize these variables so as to improve hydrogen production while minimizing energy consumption, advancing AEM technology for sustainable energy application. Their results showed that increases in electrolyte concentration, temperature, and flow rate significantly improved the hydrogen output, with the highest production being achieved at the 2.0 M KOH concentration, 60 °C, and a 9 mL/min flow rate. Under these conditions, the system produced 61.13 mL/min of hydrogen at an energy efficiency of 69.64%. This work demonstrated that fine-tuning these parameters can enhance the performance of AEM electrolysis systems, making them a more efficient method for sustainable hydrogen production. A key challenge for AEM is balancing high ionic conductivity with mechanical and alkaline stability. In another study (Contribution 7), Gao et al. introduced a facile and effective approach to develop AEMs by blending polyethylene glycol (PEG) into imidazolium-functionalized polysulfone (ImPSF) for the production of green hydrogen. This blend, referred to as ImPSF-PEGx, enhanced the membrane’s hydrophilicity and formed well-structured ion-conducting channels, promoting the transport of hydroxide ions. PEG also acted as a pore-forming agent, creating microporous structures that facilitate water uptake while maintaining dimensional stability. The blended membranes demonstrated improved water uptake, ionic conductivity, and mechanical properties compared to their non-blended counterparts. The optimal ImPSF-PEG1000 membrane exhibited superior performance, doubling the ionic conductivity of the pristine ImPSF and maintaining high stability under alkaline conditions. This research provides a cost-effective and scalable solution to improving AEM performance, which is vital for the large-scale application of green hydrogen production technologies. In a similar study (Contribution 8), Noor Azam and co-workers further presented a parametric study on the performance of polymer electrolyte membrane (PEM) electrolyzers, which were used for hydrogen production through water electrolysis. Various parameters such as current density, water flow rate, operating temperature, and anode electrocatalyst type were investigated to determine their effects on the efficiency and durability of the PEM electrolyzer. Their study identified the optimal conditions for maximizing hydrogen production while minimizing energy consumption, highlighting the importance of electrocatalyst selection and operating conditions in improving electrolyzer performance.

Membrane-based gas/liquid separation technologies have rapidly evolved as promising solutions for industrial applications, particularly in processes like CO_2_ removal and hydroformylation reactions. Molecular design plays a crucial role in enhancing the separation efficiency of polymer membranes. By introducing specific functional groups such as hydroxyl (-OH) into polymer backbones, it is possible to tailor the permeability and selectivity of membranes for specific gases and liquids. In a study (Contribution 9), Grushevenko et al. focused on the construction of a polysiloxane-based material, i.e., polydecylmethylsiloxane (PDecMS), and examined the effects of introducing hydroxyl groups into its structure on the gas permeability and liquid separation properties. The hydrophilic -OH groups were introduced via a hydrosilylation reaction, and the resulting membranes were tested for their ability to separate gases such as CO_2_, N_2_, and O_2_, as well as liquids like aldehydes and olefins. The development of these polymer membranes was motivated by the need to improve the selectivity and transport efficiency of acid gases and organic compounds. By comparing the performance of -−OH modified and unmodified PDecMS, this work was anticipated to develop advance membrane technology for industrial applications, including the decarbonization efforts and organic compound separation.

Traditional artificial skin materials often require external stimuli for their adhesion, making them unsuitable for certain biomedical applications. Contribution 10 proposed a self-powered gradient hydrogel sensor with reversible temperature-triggered adhesion for potential applications in artificial skin and biomechanical monitoring systems. The developed hydrogels, polymerized with 2-(dimethylamino)ethyl methacrylate (DMAEMA) and N-isopropylacrylamide (NIPAM), exhibited temperature-sensitive adhesion properties. At higher temperatures (above 37 °C), the hydrogel maintained high adhesion, while at room temperature, it was easily detached. This novel self-powered hydrogel offered promise for artificial skin systems and sensors.

Polyphenylene sulfone (PPSU) is known as a high-performance thermoplastic polymer with notable thermal stability, chemical resistance, and mechanical strength. Given its wide applications in industries such as automotive, aerospace, and membrane technology, optimizing the synthesis process is critical for producing PPSU with enhanced performance. Zhansitov and co-workers investigated the effects of different solvents and monomer ratios on the properties of PPSU (Contribution 11), aiming to providing a detailed understanding of how varying synthesis conditions, especially solvent choice and monomer ratios, impact the molecular weight; chemical structure; and mechanical, thermal, and rheological properties of PPSU. Specifically, the study compared the effects of dimethyl sulfoxide (DMSO), N,N-dimethylacetamide (DMAA), and N-methylpyrrolidone (NMP) as solvents, and assessed how these impact polymer stability, flow properties, and the feasibility of their use in producing filtration membranes.

The development of natural, low-cost, and biodegradable polymer membranes to reduce the reliance on petroleum-derived commercial membranes has become a pressing need in recent years. The study conducted by Musa et al. (Contribution 12) presented the use of a sodium alginate/polyvinyl alcohol (SA/PVA)-blended membrane, modified by hydrophilic montmorillonite (MMT) fillers, as a cost-effective and environmentally friendly alternative to commercial Nafion membrane for direct methanol fuel cells (DMFCs). The doping of MMT into the SA/PVA matrix improved membrane performance by enhancing proton conductivity and reducing methanol uptake. The MMT filler, at an optimal loading of 10 wt%, demonstrated the highest proton conductivity (9.38 mS/cm) and the lowest methanol uptake (89.28%) at room temperature. Overall, this research demonstrated the potential of using natural and synthetic polymer composites in developing efficient and affordable PEM for DMFCs. Starch is a promising biodegradable polymer, but it suffers from poor mechanical properties. In another study (Contribution 13), de Vilhena and co-workers investigated the mechanical properties of corn starch-based films, reinforced with sisal nanofibers and plasticized with glycerol. Sisal fibers, derived from *Agave sisalana*, were chosen due to their wide availability, low cost, and high potential as a natural fiber reinforcement. The introduction of glycerol clearly enhanced the flexibility by reducing intermolecular forces in starch, while sisal nanofibers were expected to improve the tensile strength of polymer membranes. Their findings revealed that while adding sisal nanofibers generally reduced tensile stress, the elongation of the membranes increased with higher glycerol content, making them more flexible. This study demonstrated a useful strategy for developing biodegradable packaging materials from corn starch nanocomposites, contributing to sustainable alternatives in the packaging industry.

## 3. Conclusions and Outlooks

In summary, the field of polymer membrane technology is rapidly advancing, driven by the needs for sustainable solutions to environmental challenges. Recent trends in fabrication techniques, characterization methods, and functionalization strategies are enabling the development of high-performance membranes toward specific applications. As research continues to evolve, polymer membranes will undoubtedly play an increasingly important role in addressing the global challenges of pollution, resource scarcity, and climate change.

To promote the development of advanced polymeric membranes, here, we would like to demonstrate several potential research topics in this promising field. Firstly, the fabrication of polymer membranes from sustainable, bio-based, and biodegradable polymers, such as polylactic acid (PLA) or natural biomass polymers like chitosan and cellulose, is highly encouraged to minimize environmental impact while maintaining or enhancing membrane performance. Secondly, the development of advanced fabrication techniques such as 3D printing, molecular imprinting, or nano-manufacturing techniques is necessary to produce highly uniform, customizable membranes with precise control over pore size, structure, and functionality, enabling improved selectivity and permeability of the resultant membranes. Thirdly, the incorporation of smart functional materials into polymer membranes that can respond to external stimuli (e.g., pH, temperature, or light) is another research direction since many polymer membranes lack adaptability in changing environmental conditions. Functionalizing membranes with stimuli-responsive polymers could allow them to adapt to environmental changes, optimizing the filtration or adsorption processes dynamically. Fourthly, it is urgent to explore novel surface modifications and coatings that render anti-fouling or self-cleaning properties for polymer membranes. For instance, incorporating hydrophilic or zwitterionic polymers, or even bioinspired designs mimicking lotus leaf structures, can enhance membrane performance by reducing fouling and enabling self-regeneration. Fifthly, the exploration of energy-efficient membrane processes, such as forward osmosis or pressure-retarded osmosis, can reduce energy consumption while treating polluted water. Moreover, integrating membranes with renewable energy sources like solar or wind to create off-grid, low-energy environmental remediation systems is encouraged. Finally, it is suggested to use comprehensive lifecycle assessments (LCAs) to quantify the environmental benefits and drawbacks of various polymer membrane types. Research should also focus on developing strategies for the sustainability of polymer membrane, including recycling, reuse, and safe disposal to reduce the long-term environmental impacts.
